# Association between a high triglyceride–glucose index and chronic kidney disease in adult patients with latent autoimmune diabetes

**DOI:** 10.1186/s12902-023-01465-5

**Published:** 2023-09-28

**Authors:** Xiuli Fu, Zihui Xu, Qin Tan, Wei Wei, Zhongjing Wang

**Affiliations:** Department of Endocrinology, The Central Hospital of Wuhan, Tongji Medical College, Huazhong University of Science and Technology, No. 26 of Shengli Street, Jiangan District, Wuhan, 430061 China

**Keywords:** Triglyceride–glucose index, Latent autoimmune diabetes in adults, Insulin resistance, Chronic kidney disease

## Abstract

**Background:**

Insulin resistance (IR) is one of the risk factors for chronic kidney disease (CKD) and diabetes. The triglyceride–glucose (TyG) index is considered a reliable alternative marker of IR. We investigated the correlation between the TyG index and the severity of CKD in patients with latent autoimmune diabetes in adults (LADA).

**Methods:**

This cross-sectional study included 288 patients with LADA in the department of endocrinology at our hospital between January 2018 and January 2022. The TyG index was calculated as Ln [TG (mg/dl) × fasting blood glucose (FBG) (mg/dl) / 2]. All individuals were divided into either a LADA + CKD group or a LADA + non-CKD group according to the presence or absence of CKD. A correlation analysis, logistic regression analysis and receiver operating characteristics curve analysis were performed.

**Results:**

A total of 130 (45.1%) participants were identified as having CKD. Compared with the non-CKD group, the CKD group had a longer disease duration and a higher proportion of smokers; patients were more likely to have hypertension and higher serum creatinine, triglyceride, cholesterol, low-density lipoprotein cholesterol, FBG, uric acid estimated glomerular filtration rates (eGFR) and TyG levels as well as lower high-density lipoprotein cholesterol levels (all *P* < 0.05). The positive relationship between the TyG index and the urinary albumin/creatinine ratio was significant (*r* = 0.249, *P* = 0.010). There was also a significant correlation between the TyG index and the eGFR (*r* = − 0.211, *P* = 0.034) after adjusting for confounding factors. The area-under-the-curve value of the TyG index was 0.708 (95% confidence interval: 0.61–0.81, *P* < 0.001).

**Conclusions:**

The TyG index is significantly associated with the severity of CKD in patients with LADA. This conclusion supports the clinical application of the TyG index for the assessment of kidney disease in patients with LADA.

## Background

Latent autoimmune diabetes in adults (LADA) is a type of adult-onset autoimmune diabetes that reflects the clinical characteristics of type 2 diabetes and the presence of type 1 diabetes-related autoantibodies. The clinical and genetic features of the disease are intermediate between those of typical type 1 diabetes mellitus (T1DM) and type 2 diabetes mellitus (T2DM), and it is labelled as ‘slowly evolving immune-mediated diabetes’ by the World Health Organization (WHO) [1]. Epidemiological studies show that LADA may account for 2–12% of all cases of diabetes in the adult population. It is a heterogeneous form of diabetes with a pathogenesis that includes the autoimmune destruction of pancreatic beta cells as well as a degree of insulin resistance (IR) [2].

The triglyceride–glucose (TyG) index, comprising fasting plasma glucose and triglycerides (TGs), is significantly associated with the hyperinsulinemia-normal glucose clamp test and the homeostasis model assessment of IR (HOMA-IR) [3]. A large number of studies have demonstrated that the TyG index, which outperformed the HOMA-IR, is correlated with the incidences of coronary heart disease, hypertension, myocardial infarction and other cardiovascular diseases [4–6]. Given that HOMA-IR confers a high risk of kidney disease, it is plausible that the TyG index may be implicated in the pathogenesis and progression of kidney disease in patients with diabetes. Recent studies confirmed that the TyG index could predict kidney disease in subjects with type 2 diabetes [7]. However, there are currently no such data for patients in the LADA category. Therefore, the present study aimed to determine the association between the TyG index and the severity of chronic kidney disease (CKD) in patients with LADA.

## Methods

### Participants and study design

This cross-sectional study included 288 patients with LADA at our hospital between January 2018 and January 2022. All individuals were divided into either a LADA + CKD group or a LADA + non-CKD group according to the presence or absence of CKD. The inclusion criteria for patients with LADA were as follows [8]: (1) adult age of onset (> 30 years); (2) diagnosed with diabetes (WHO diagnostic criteria) [1]; (2) no ketoacidosis in the first 6 months after a diagnosis of diabetes; (3) no insulin requirement for at least 6 months after diagnosis; (4) generalised kidney disease autoantibody-positive and (5) fasting C-peptide > 0.2 ng/ml.

The exclusion criteria were as follows: (1) participants with secondary diabetes, those who were pregnant and those with a malignancy; (2) participants in a state of ketoacidosis; (3) participants with a previous history of using statins or TG-lowering drugs; (4) participants who had used immunosuppressive drugs, antibiotics or steroid medication in the preceding 3 months; (5) patients with other severe diseases and (6) those with a lack of necessary laboratory or physical examination data.

### Data collection and measurements

For the included patients, clinical data were extracted from electronic medical records, including age, sex, duration of LADA, height, weight, hypertension, systolic blood pressure (SBP), diastolic blood pressure (DBP), lipid profile and glucose metabolism indices. Blood samples were collected from the patients after fasting for 8 h. All blood and urine specimens were tested immediately after collection. Biomedical measurements were taken for serum or plasma separation upon blood collection. Overnight fasting blood samples were collected from each participant to test for fasting blood glucose (FBG), serum total cholesterol (TC), low-density lipoprotein cholesterol (LDL-C), high-density lipoprotein cholesterol (HDL-C), TGs, serum uric acid (UA), glycosylated haemoglobin A1c (HbA1c) and liver/renal functions. Serum C-peptide levels were detected by a chemiluminescent immunometric assay (Roche Diagnostics Ltd.). Urinary albumin and serum creatinine (Scr) were measured using laser immunonephelometry (Roche Diagnostics Ltd.). The serum levels of interleukin-10 (IL-10), tumour necrosis factor-α (TNF-α), interleukin-6 (IL-6) and interleukin-1β (IL-1β) were quantified using the following enzyme-linked immunosorbent assay (ELISA) kits (all from Abcam, USA) in accordance with the manufacturer’s instructions: Human IL-10 ELISA Kit (ab46034), Human TNF-α ELISA Kit (ab181421), Human IL-6 ELISA Kit (ab178013), Human IL-1β ELISA Kit (ab100562). The optical density of the samples was determined at 450 nm using an ImmunoChem-2100 microplate reader (HTI Ltd., USA).

Definition of variables: Hypertension was defined as SBP ≥ 140 mmHg and/or DBP ≥ 90 mmHg following repeated examination or a prior diagnosis of hypertension by a physician. The estimated glomerular filtration rates (eGFRs) were calculated using the Chronic Kidney Disease Epidemiology Collaboration formula [9]. The urinary albumin/creatinine ratio (UACR) was calculated as the UACR. Chronic kidney disease was defined as UACR ≥ 30 mg/g and/or eGFR < 60 ml/min/1.73m^2^ [10]. Body mass index was calculated as the patient’s weight in kilograms divided by the square of their height in metres. The TyG index was defined using the formula Ln [TG (mg/dl) × FBG (mg/dl)] / 2 [11].

### Statistical analysis

All analyses were performed using SPSS 26.0 software (IBM Statistics Group). The clinical characteristics of all the participants were described using means ± standard deviations for continuous variables and percentages for categorical variables. Normally distributed data were analysed using the Student’s *t* test or a one-way analysis of variance, with Bonferroni corrections for post hoc analysis. Non-normally distributed data were analysed using the Mann–Whitney test or the Kruskal–Wallis H test to identify statistical differences between groups. Categorical variables described the number and percentage of each type, and comparisons between groups were processed using Chi-squared or Fisher’s exact tests. Pearson’s correlation and partial correlation analyses were performed for the correlation analysis of the TyG index with other variables. Univariate and multivariate logistic regression analyses were performed to determine the effect of different TyG levels on the severity of CKD. Receiver operating characteristic curve (ROC) analyses were conducted to determine the severity of CKD in participants. A value of P < 0.05 was considered statistically significant.

## Results

### Characteristics of patients with latent autoimmune diabetes with and without chronic kidney disease

**Fig. 1:**
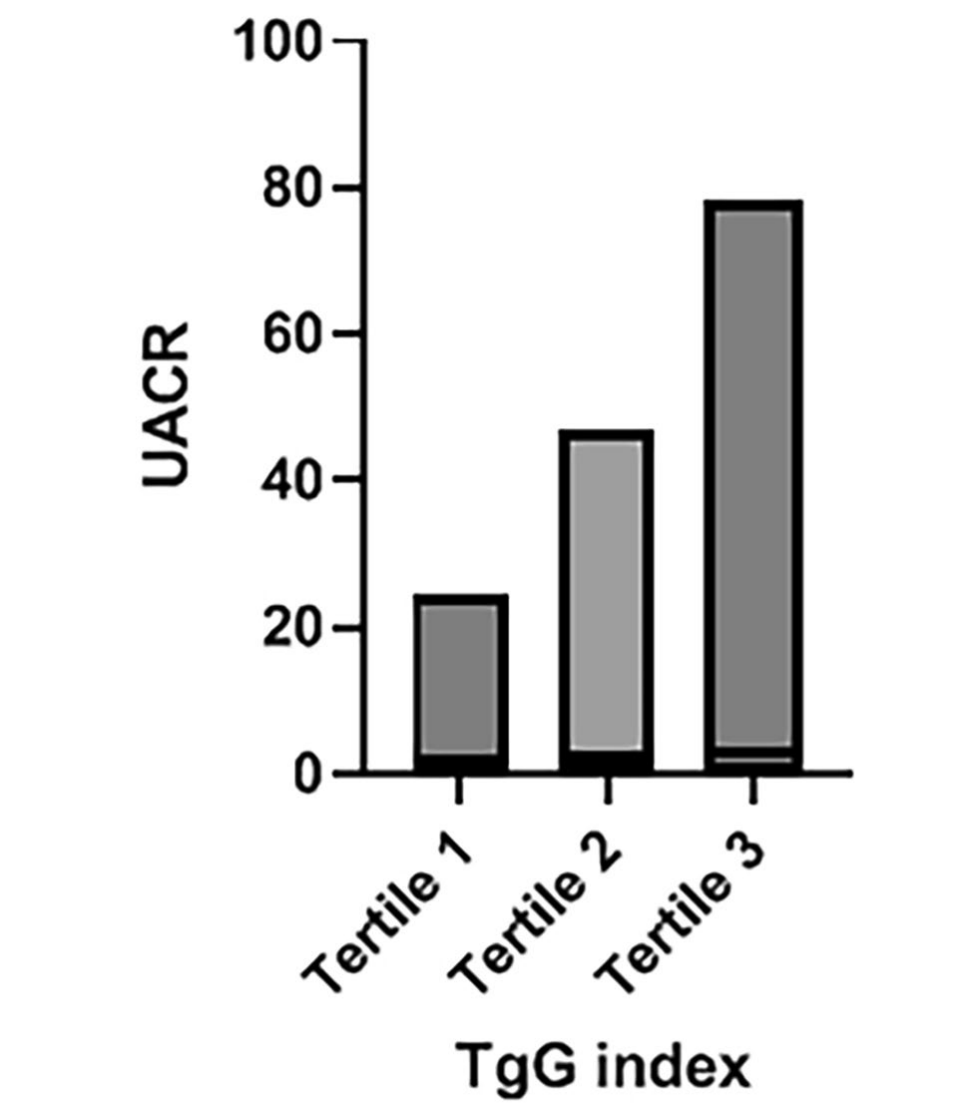
The relationship of UACR and TyG in LADA patients

A total of 288 individuals participated in this study, in which men accounted for 51.3% and women 48.7%; 130 patients were included in the LADA + CKD group and 158 in the LADA + non-CKD group. Table 1 shows the demographic and clinical characteristics of the two groups. The two participant groups did not differ in terms of gender, age and fasting C-peptide levels. Compared with the patients without CKD, those with CKD had a longer disease duration, and a higher proportion were smokers, who were more likely to have hypertension; they also had higher mean Scr, TG, TC, LDL-C, FBG, HbA1c, UA, eGFR and TyG levels and lower HDL-C levels (all *P* < 0.05). There was no difference in the expression levels of inflammation-related factors (IL-10, TNF-α, IL-6 and IL-1β) (all *P* > 0.05) (see Table 1; Fig. 1).

**Table 1:** Characteristics of LADA patients with and without CKD

	LADA + non-CKD group (n = 158)	LADA + CKD group (n = 130)	P
Gender (male)	77 (48.7)	71 (54.6)	0.617
Age (years)	55.44 ± 11.55	59.18 ± 13.42	0.118
Smoke [n (%)]	16 (10.1%%)	32 (24.6%)	< 0.001
Disease duration (years)	5.34 ± 5.67	8.10 ± 5.41	0.011
BMI (kg/m^2^)	22.3 ± 3.6	23.1 ± 3.9	0.524
Hypertension [n (%)]	19 (12%)	36 (27.7%)	< 0.001
SBP (mmHg)	132.5 ± 16.3	135.9 ± 17.2	0.109
DBP (mmHg)	69.3 ± 10.2	69.4 ± 11.5	0.359
ALT (U/L)	20.15 ± 9.53	34.13 ± 25.50	< 0.001
AST (U/L)	20.57 ± 7.93	32.91 ± 21.49	< 0.001
Scr (mmol/L)	58.22 ± 11.78	101.22 ± 27.61	< 0.001
TC (mmol/L)	4.26 ± 1.09	5.18 ± 1.08	< 0.001
TG (mmol/L)	2.00 ± 1.36	2.55 ± 1.53	0.049
HDL-C (mmol/L)	1.10 ± 0.21	1.01 ± 0.15	0.010
LDL-C (mmol/L)	2.69 ± 0.91	3.37 ± 0.91	< 0.001
IL-10 (pg/mL)	1.32 ± 0.11	1.44 ± 0.20	0.057
TNF-α (pg/mL)	5.60 ± 0.78	5.44 ± 0.65	0.075
IL-6 (pg/mL)	1.61 ± 0.32	1.69 ± 0.41	0.064
IL-1β (pg/mL)	2.67 ± 0.44	2.47 ± 0.29	0.051
UA	317.20 ± 87.24	384.2 ± 128.68	0.002
FBG (mmol/L)	9.76 ± 4.58	12.84 ± 4.47	0.001
HbA1c	8.86 ± 2.45	9.75 ± 2.12	0.047
Fasting C-peptide	1.25 ± 1.11	1.37 ± 1.23	0.584
UACR (mg/g)	8.13 ± 1.46	132.56 ± 70.36	< 0.001
TyG index	8.54 ± 0.74	9.11 ± 0.78	< 0.001
eGFR	79.9 ± 13.1	48.9 ± 14.7	0.001

Note: LADA, latent autoimmune diabetes in adults; CKD, chronic kidney disease; BMI, body mass index; SBP, systolic blood pressure; DBP, diastolic blood pressure; ALT, alanine transaminase; AST, aspartate amino transferase; Scr, serum creatinine; TC, serum total cholesterol; TG, triglycerides; HDL-C, high-density lipoprotein cholesterol; LDL-C, low-density lipoprotein cholesterol; IL-10, interleukin-10; TNF-α: tumor necrosis factor-α; IL-6, interleukin-6; IL-1β, interleukin-1β; UA, serum uric acid; FBG, fasting blood glucose; HbA1c, glycosylated hemoglobin A1c; UACR, urinary albumin/creatinine ratio; eGFR, estimated glomerular filtration rate; TyG index, the triglyceride-glucose index

### Clinical and biochemical characteristics by triglyceride–glucose index

To understand the association between different TyG levels and UACRs in patients with LADA, we divided the participants into terciles according to the TyG index (*n* = 96 in each group, TyG index range of tercile 1 < 8.5; TyG index range of tercile 2 8.50–9.23; TyG index range of tercile 3 > 9.23).

The average TyG indices in the three tertile groups were 7.92 ± 0.35, 8.78 ± 0.21 and 9.69 ± 0.46. With an increase in the TyG index, the UACR of the patients in the three groups also increased significantly, indicating a possible positive correlation between the UACR and the TyG index (Fig. 1). There were statistically significant differences between the three groups concerning the number of smokers, disease duration, alanine aminotransferase, aspartate aminotransferase, Scr, TG, TC, LDL-C, HDL-C, FBG, HbA1c, UACR, TyG index and eGFR (all *P* < 0.05) (see Table 2).

**Table 2:** Demographic and clinical characteristics of participants by TyG index

	Tertile 1 (n = 96)	Tertile 2 (n = 96)	Tertile 3 (n = 96)	*P*
Gender (male)	44(45.8)	49(51.0)	55(57.3)	0.655
Age (years)	56.32 ± 12.75	58.08 ± 11.33	56.97 ± 13.63	0.832
Smoke [n (%)]	9 (9.4%)	14 (14.6%)	25 (26%)*#	< 0.001
Disease duration (years)	5.09 ± 5.99	5.88 ± 4.71	8.80 ± 5.75*#	0.012
BMI (kg/m^2^)	22.5 ± 3.7	22.9 ± 3.8	22.8 ± 3.6	0.612
Hypertension [n (%)]	16 (16.7%)	18 (18.8%)	21 (21.9%)	0.512
SBP (mmHg)	133.7 ± 16.2	135.2 ± 16.9	138.1 ± 17.3	0.063
DBP (mmHg)	68.9 ± 10.5	69.4 ± 11.2	69.7 ± 12.1	0.071
ALT (U/L)	19.04 ± 8.45	23.26 ± 16.94*	37.05 ± 25.40*#	< 0.001
AST (U/L)	19.85 ± 6.41	22.62 ± 10.87*	35.91 ± 23.21*#	< 0.001
Scr (mmol/L)	63.43 ± 18.86	74.17 ± 21.54*	95.18 ± 36.40*#	< 0.001
TC (mmol/L)	4.17 ± 0.86	4.78 ± 1.19	5.07 ± 1.28*#	0.003
TG (mmol/L)	1.12 ± 0.39	2.03 ± 0.86*	3.60 ± 1.53*#	< 0.001
HDL-C (mmol/L)	1.12 ± 0.39	1.06 ± 0.19	0.99 ± 0.16*#	0.009
LDL-C (mmol/L)	2.65 ± 0.88	2.99 ± 0.89	3.33 ± 1.02*#	0.017
UA	331.59 ± 90.47	343.68 ± 110.23	366.86 ± 132.87	0.394
FBG (mmol/L)	8.09 ± 3.28	10.77 ± 4.04*	14.59 ± 4.52*#	< 0.001
HbA1c	8.31 ± 2.15	8.97 ± 1.96	10.50 ± 2.38*#	< 0.001
Fasting C-peptide	1.27 ± 1.02	1.32 ± 1.16	1.32 ± 1.35	0.982
UACR (mg/g)	6.58 ± 3.59	76.75 ± 11.77*	174.22 ± 35.37*#	< 0.001
TyG index	7.92 ± 0.35	8.78 ± 0.21	9.69 ± 0.46*#	< 0.001
eGFR	77.9 ± 15.3	68.4 ± 17.2*	53.7 ± 16.7*#	< 0.001

Note: tertile 1: TyG index < 8.5; tertile 2: 8.50 ≤ TyG index ≤ 9.23; tertile 3: TyG index > 9.23. LADA, latent autoimmune diabetes in adults; CKD, chronic kidney disease; BMI, body mass index; SBP, systolic blood pressure; DBP, diastolic blood pressure; ALT, alanine transaminase; AST, aspartate amino transferase; Scr, serum creatinine; TC, serum total cholesterol; TG, triglycerides; HDL-C, high-density lipoprotein cholesterol; LDL-C, low-density lipoprotein cholesterol; UA, serum uric acid; FBG, fasting blood glucose; HbA1c, glycosylated hemoglobin A1c; UACR, urinary albumin/creatinine ratio; eGFR, estimated glomerular filtration rate; TyG index, the triglyceride-glucose index

*: compared with Tertile 1, p < 0.05; #: compared with Tertile 2, p < 0.05

### Association between triglyceride–glucose index and chronic kidney disease in the multivariate analysis

Based on the multivariate logistic regression analysis (Table 3), the TyG index was independently related to CKD in patients with LADA after adjusting for age, sex, disease duration, body weight, the presence of hypertension and serum UA.

**Table 3:** Odds ratio of CKD in LADA patients

Variable	B value	Odds ratio	95%CI	*P*-value
TyG index	1.287	2.465	1.291–4.708	0.006
Disease duration	0.876	1.005	0.972–1.146	0.197
Hypertension	3.221	5.349	2.112–13.55	0.598
UA	0.127	1.005	1.001–1.009	0.328

Note: LADA, latent autoimmune diabetes in adults; CKD, chronic kidney disease; TyG index, the triglyceride-glucose index; UA, serum uric acid

### Correlation of triglyceride–glucose index with urinary albumin/creatinine ratio and estimated glomerular filtration rates

We investigated the association between the TyG index, UACR and eGFR. Based on the correlation analysis, the TyG index positively correlated with the UACR (*r* = 0.319, *P* = 0.001). After adjusting for age, sex, disease duration, body weight, serum UA and the presence of hypertension, the positive relationship between the TyG index and the UACR remained significant (*r* = 0.249, *P* = 0.010). There was also a significant correlation between the TyG index and the eGFR (*r* = − 0.309, *P* = 0.001). After adjusting for confounding factors, a moderate relationship was identified between the TyG index and the eGFR (*r* = − 0.211, *P* = 0.034) (see Table 4).

**Table 4:** Correlation of TyG index with UACR and eGFR in patients with LADA

TyG index	UACR		eGFR	
	Before adjusting	After adjusting	Before adjusting	After adjusting
*r*-value	0.319	0.249	-0.309	-0.211
*P*-value	0.001	0.010	0.001	0.034

Note: LADA, latent autoimmune diabetes in adults; TyG index, the triglyceride-glucose index; UACR, urinary albumin/creatinine ratio; eGFR, estimated glomerular filtration rate

### Receiver operating curve analysis for determination of the severity of chronic kidney disease

**Fig. 2:**
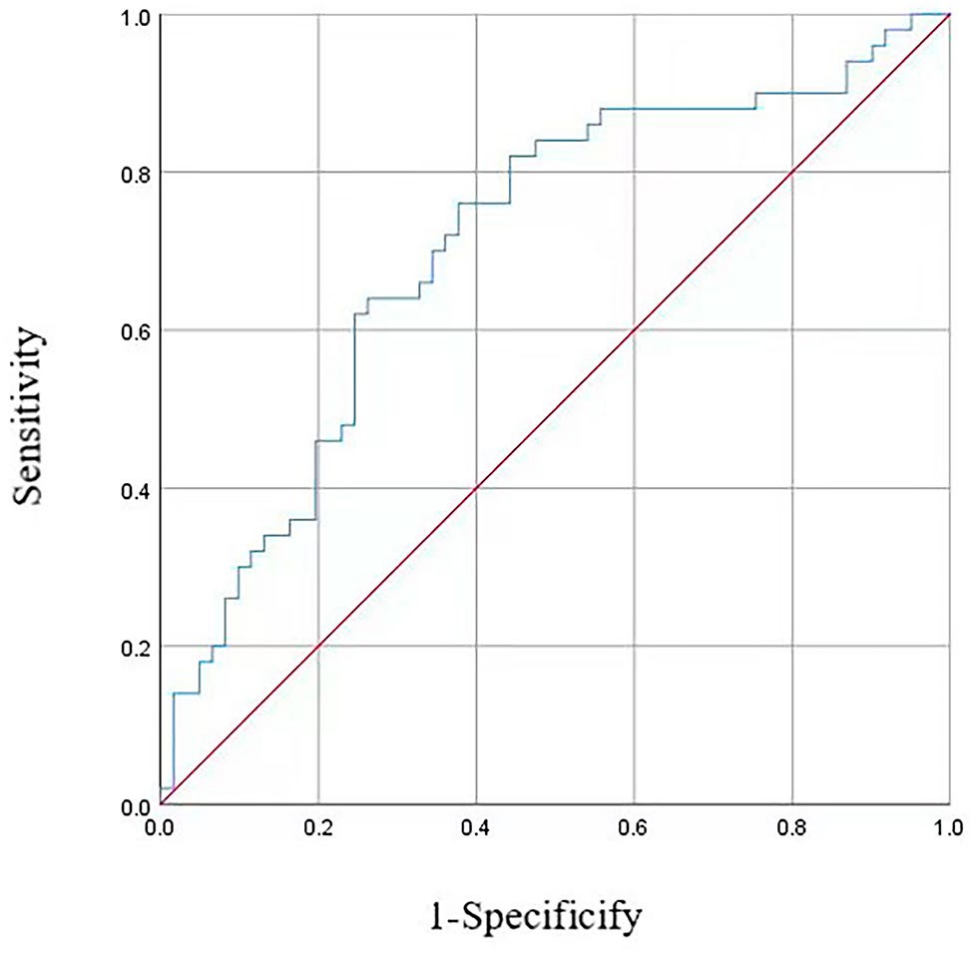
ROC analyses of TyG index

The ROC analysis was performed to evaluate the performance of the TyG index to identify patients with a risk of CKD. The area-under-the-curve value of the TyG index was 0.708 (95% confidence interval: 0.61–0.81, *P* < 0.001) (Fig. 2).

## Discussion

The mechanisms for the association between the TyG index, LADA and kidney disease have not yet been clarified, but they are believed to include HOMA-IR, autoimmune, inflammation, lipid and glucose disorders [5]. The TyG index not only reflects IR but is also closely associated with these mechanisms, which may explain why a considerable number of participants with LADA and an increased TyG index ultimately developed kidney disease. Our results suggest that the TyG index is significantly associated with the severity of CKD in patients with LADA.

Latent autoimmune diabetes in adults is a well-recognised diabetes sub-type of immune-mediated type 1 diabetes. Previous studies revealed that patients with LADA displayed mixed innate immune cell features of T1DM and T2DM [8]. Autoimmune attacks against islet β cells and IR caused by chronic systemic inflammation are both involved in the LADA disease process.

Podocytes are insulin-sensitive epithelial cells that create slit diaphragms within glomeruli to ensure proper filtration. Podocyte injury is a critical factor in the progression of kidney disease. Studies have shown that the inability of podocytes to respond to insulin may partially account for the decreased number of podocytes observed in early diabetic nephropathy in diabetic mice [12]. Rousseau et al. found that a diabetes-induced reduction of dual-specificity phosphatase 4 leads to the activation of natural killer (NK) cells and elevated Nox4 expression, which contributes to podocyte dysfunction, IR and the progression of kidney disease [13]. These abnormal physiological processes are common in patients with LADA and appear to partly explain the high risk of kidney injury in patients with both LADA and an increased TyG index. However, this study did not show significant differences in serum inflammatory biomarkers in patients with LADA with or without CKD, which may be because the study did not further divide subgroups according to different CKD stages for comparative analysis.

Latent autoimmune diabetes in adults is an autoimmune-mediated type of diabetes, and T lymphocytes are a key factor in its pathogenesis [14]. Data demonstrates that, based on these T-cell subset alterations, IR may be involved in the pathogenesis of LADA [15]. Positive correlations between the serum levels of autoantibodies and pancreatic islet dysfunction in the autoimmune disease model of mice may partially reflect the relationship between autoimmunity and LADA [16]. Islet-reactive T cells generated in the pancreas migrate to metabolically active tissue, such as kidney tissue, where they induce IR either by sustaining chronic tissue inflammation or targeting protective elements for the development of IR. Studies have revealed that many patients with diabetes were found to have autoimmune abnormality-related renal diseases, such as tubule interstitial nephritis, immunoglobulin A nephropathy and membranous proliferative glomerulonephritis [17, 18]. In diabetic kidney disease related to autoimmune nephritis, Hiramatsu et al. found that hyperglycaemia modified its pathology by decreasing the mesangial area and aggravating tubulointerstitial lesions, which were mainly observed as an injury in renal tubular epithelial cells [19].

During the initiation and progression of LADA, macrophages, NK cells, dendritic cells and neutrophils play a crucial role [20]. Studies found an increased amount of inducible interferon (IFN)-g (+) NK cells in patients with newly diagnosed LADA. Additionally, IFN-g released by NK cells may promote the development of LADA by affecting islet β cells and is closely associated with IR [21, 22]. A large number of lymphocytes, macrophages and mast cells accumulate in the kidney tissue of patients with diabetic kidney disease and secrete large amounts of inflammatory mediators, cytokines and oxygen free radicals, directly or indirectly leading to IR and kidney damage [23–26]. Metabolic disorders in patients with LADA activate inflammatory signals in the body, which, in turn, causes the deposition of extracellular matrixes in the kidney and promotes fibrosis. Yu et al. found that neutrophil counts were closely associated with kidney disease in patients with LADA and suggested that neutrophil-mediated inflammation may be involved in the pathogenesis of kidney disease in cases of LADA [27].

Insulin resistance plays a crucial role in the occurrence of kidney disease in patients with LADA; at the same time, autoimmune damage, inflammation and glucose and lipid metabolism disorders promote the process. Pathological processes can result in increased TG and glucose levels, thus accelerating TyG levels, indicating that the TyG index is related to the occurrence of kidney disease in patients with LADA and is representative of disease severity. Glucose and lipid metabolism disorders in patients with LADA promote an increased IR; accordingly, IR-induced inflammation and immune response appear to be fundamentally linked to kidney disease in patients with LADA.

Our study provides evidence for the association between the TyG index and kidney disease in patients with LADA despite the adjustments needed for traditional risk factors, such as age, smoking, disease duration, hypertension and UA. We showed that patients with LADA had an increased risk of kidney disease and a higher TyG index. In this study, TyG was used to emphasise the important role of IR in the occurrence of kidney disease in patients with LADA, providing a new way to indicate both LADA and the severity of kidney disease among at-risk patients.

Our study has several limitations. First, the sample size of patients with LADA was relatively small, and its retrospective cross-sectional single-centre design may have caused bias. Future large-scale prospective studies are required to further clarify the predictive power of the TyG index on kidney disease risk among patients with LADA. Second, patients with severe kidney disease were not included in this study, which may have led to an underestimation of the efficacy of the TyG index in predicting kidney disease. Future studies should consider dividing subgroups according to the stages of CKD to further validate and generalise the conclusions of this study. Third, our study was based on a single TyG measurement, and the potential cardiovascular implications of changes in TyG (for example, in response to changes in lifestyle or drug therapy) over time require further investigation.

In conclusion, the TyG index is significantly associated with the severity of CKD in patients with LADA. The TyG index can be used as a supplement to the classic risk factors for CKD among patients with LADA, and the association may be higher and reflect lesion severity to some extent. This study supports the clinical application of the TyG index in evaluating kidney disease in the LADA population.

## Data Availability

All data generated or analyzed during this study are included in this published article.
